# Standardized Preclinical *In Vitro* Blood–Brain Barrier Mouse Assay Validates Endocytosis-Dependent Antibody Transcytosis Using Transferrin-Receptor-Mediated Pathways

**DOI:** 10.1021/acs.molpharmaceut.2c00768

**Published:** 2023-02-21

**Authors:** Jamie I. Morrison, Alex Petrovic, Nicole G. Metzendorf, Fadi Rofo, Canan U. Yilmaz, Sofia Stenler, Hanna Laudon, Greta Hultqvist

**Affiliations:** † Institutionen för Farmaci, Uppsala Universitet, Uppsala 752 37, Sweden; ‡ BioArctic AB, Stockholm 112 51, Sweden

**Keywords:** *in vitro* BBB, blood−brain barrier, transferrin receptor, TfR, transcytosis, 3R

## Abstract

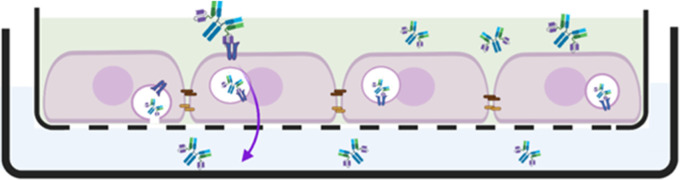

The presence of the blood–brain barrier (BBB) creates a nigh-on impenetrable obstacle for large macromolecular therapeutics that need to be delivered to the brain milieu to treat neurological disorders. To overcome this, one of the strategies used is to bypass the barrier with what is referred to as a “Trojan Horse” strategy, where therapeutics are designed to use endogenous receptor-mediated pathways to piggyback their way through the BBB. Even though *in vivo* methodologies are commonly used to test the efficacy of BBB-penetrating biologics, comparable *in vitro* BBB models are in high demand, as they benefit from being an isolated cellular system devoid of physiological factors that can on occasion mask the processes behind BBB transport via transcytosis. We have developed an *in vitro* BBB model (In-Cell BBB-Trans assay) based on the murine cEND cells that help delineate the ability of modified large bivalent IgG antibodies conjugated to the transferrin receptor binder scFv8D3 to cross an endothelial monolayer grown on porous cell culture inserts (PCIs). Following the administration of bivalent antibodies into the endothelial monolayer, a highly sensitive enzyme-linked immunosorbent assay (ELISA) is used to determine the concentration in the apical (blood) and basolateral (brain) chambers of the PCI system, allowing for the evaluation of apical recycling and basolateral transcytosis, respectively. Our results show that antibodies conjugated to scFv8D3 transcytose at considerably higher levels compared to unconjugated antibodies in the In-Cell BBB-Trans assay. Interestingly, we are able to show that these results mimic *in vivo* brain uptake studies using identical antibodies. In addition, we are able to transversely section PCI cultured cells, allowing for the identification of receptors and proteins that are likely involved in the transcytosis of the antibodies. Furthermore, studies using the In-Cell BBB-Trans assay revealed that transcytosis of the transferrin-receptor-targeting antibodies is dependent on endocytosis. In conclusion, we have designed a simple, reproducible In-Cell BBB-Trans assay based on murine cells that can be used to rapidly determine the BBB-penetrating capabilities of transferrin-receptor-targeting antibodies. We believe that the In-Cell BBB-Trans assay can be used as a powerful, preclinical screening platform for therapeutic neurological pathologies.

## Introduction

The blood–brain barrier (BBB) is one of the most tightly regulated physiological interfaces, regulated by physical, transport, and metabolic barrier mechanisms to maintain the proper influx and efflux of metabolites to and from the brain.^1^ The impermeable nature of the BBB importantly regulates the neuronal signaling microenvironment but at the same time hinders the delivery of therapeutic agents. Aside from invasively delivering therapeutics to the neuronal microenvironment, current systemic delivery approaches typically rely on therapeutic interventions that can readily diffuse across the BBB and are no larger than 400 Da in size^2^ or the blood–cerebrospinal fluid barrier (BCSFB), whose potential for drug delivery to the brain is currently the focus of multiple studies.^3^ This of course hampers and limits treatment strategies aimed at tackling neuronal disorders.

Many researchers worldwide are trying to find effective, safe therapeutic avenues to circumvent the molecular pathology evident in neurodegenerative diseases and other brain diseases. Protein-based biological drugs are the fastest-growing field in drug development, with a quarter of the newly approved drugs being proteins. Protein-based biologics are uniquely adept in binding specifically to a disease target, which enables them to treat diseases that small molecules cannot treat.^4^ The most recognized strategy to shuttle large biologics across the selectively permeable endothelial cell layer is to use the receptor-mediated endocytosis/transcytosis (RMT) pathways of the BBB.^5^ The methodology relies on discovering receptors found on the apical side of the endothelial cell unit of the BBB that normally regulate the transport of essential nutrients and growth factors from the blood into the brain. Artificial, protein-based transporters that bind to these receptors can then be designed, and these can in the ideal situation cross the endothelium via endocytosis/transcytosis into the extracellular environment of the brain. Since the endosome is large, one can recombinantly link therapeutic payloads to these transporters. We have successfully used such “Trojan Horse” strategies to deliver intravenously injected antibodies against the pathological Aβ protofibrils to the Alzheimer’s diseased brain in mice.^6^ Our transporter bound the transferrin receptor (TfR) that is expressed promiscuously on the apical membrane of the endothelial cells, which is responsible for the transport of transferrin and iron to the brain parenchyma. The uptake compared to the antibody without the transporter was increased approximately 80 times. The same transporter has also been employed to to efficiently transport both antibody fragments and peptides into the brain.^7−10^ When the antibody is used to transport therapeutic or diagnostic protein payloads, clear therapeutic effects and diagnostic images have been observed.^6^ It has also been shown, by capillary depletion studies, that most of the proteins transported with scFv8D3 reaches the brain parenchyma, with a small proportion attached to or inside the endothelial cells.^11^

Alongside the advancement of novel methods to noninvasively deliver therapeutics to the brain, new preclinical *in vitro* analytical methodologies also need to be developed, not only to complement the necessary *in vivo* brain shuttling efficacy studies of the therapeutic in question but also to abide by the directives of the EU concerning the reduction, replacement, and refinement of animals used in research.^12^ The development of an *in vitro* cell culture model that mimics the *in vivo* blood–brain barrier could be one such technique. It allows the user to pulse or load a therapeutic in the apical chamber of a cell-coated permeable support membrane (PCI), followed by the collection and analysis of media in the basolateral chamber at various time points (referred to as the chase). In this setup, the apical chamber is a static representation of the luminal blood flow seen in the arterial and venous capillaries of the BBB, whereas the basolateral compartment resembles the abluminal brain milieu. Ideally, if the therapeutic does have brain shuttling properties, its presence should be detectable within the basolateral chamber during the chase phase of the *in vitro* assay. There are several *in vitro* BBB model systems of varying complexity that are under development, which can be employed to mimic the *in vivo* BBB in certain physiological settings.^13^ However, the majority of the published *in vitro* BBB cell culture systems are focused on creating a model that closely resembles the *in vivo* BBB rather than creating a cell culture system that can be used to test the brain shuttling efficacy of therapeutics. Simplified *in vitro* models of the human BBB, used to assess the transcytosis rates of antibodies directed against potential brain shuttle receptors, have been developed.^14,15^ They are based upon an *in vitro* protocol developed using primary cultures of bovine brain microvessel endothelial cells,^16^ which address the problem with leakiness of the *in vitro* system by instigating a washing regime that results in only analyzing what has entered the cells. However, there is a lack of simple, descriptive mouse *in vitro* BBB systems that can be used pre-emptively to assess the efficacy of protein-based BBB-penetrating therapeutics. The Immortalized Mouse Cerebral Capillary Endothelial Cell line (cEND) is an easily accessible and easy-to-handle cell murine BBB cell line that has not been previously used for the quantification of TfR-mediated transcytosis.^17,18^

**Figure 1:**
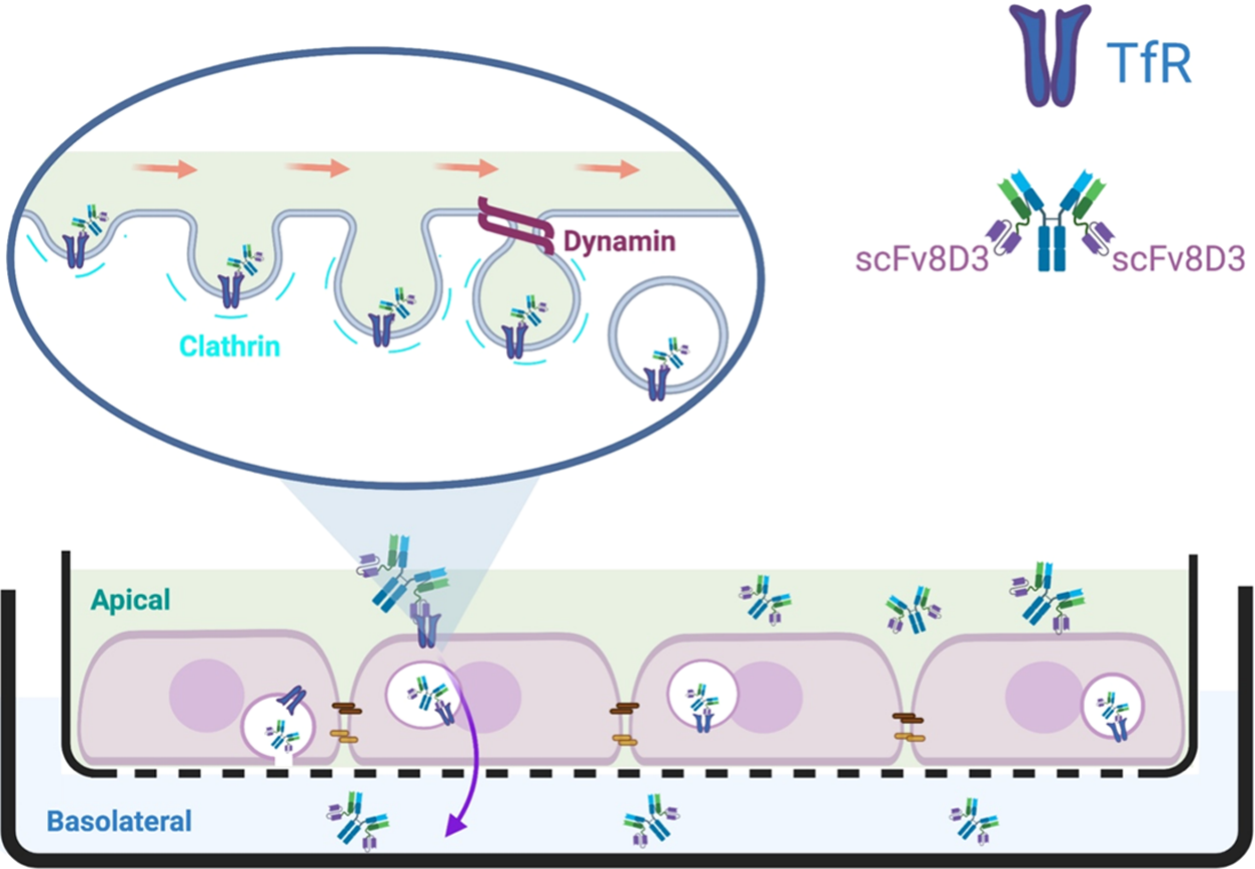
Cartoon summary depicting a single PCI setup of the In-Cell BBB-Trans assay. The cartoon shows a monolayer of mouse endothelial cells being pulsed with transferrin-receptor-binding scFv8D3-conjugated bivalent antibodies. The antibodies can be seen binding to the transferrin receptor (TfR), undergoing endocytosis into the cell (the process of which is visualized in the magnified image inset), and traveling from the apical (blood mimicking) compartment to the basolateral (brain milieu mimicking) compartment via transcytosis pathways.

Using this cell line, we have developed the In-Cell BBB-Trans assay, a standardized mouse monolayer PCI culture system that can be used to assess the brain shuttling properties of antibodies conjugated to the transferrin receptor binder 8D3, which has been shown on numerous occasions to be an excellent BBB transporter in mice.^6,19,20^ The cell culture assay is streamlined utilizing a “pulse-chase” strategy, so that the entire assay can be completed within 4 days, from initial plating of the cells to analysis. In addition, a highly sensitive enzyme-linked immunosorbent assay (ELISA) has been developed to work alongside the assay, detecting antibodies in cell medium down to a concentration as low as 0.5 pM. Furthermore, using transverse cryosections of the PCI membranes and subsequent immunohistochemical staining techniques affords the user the ability to detect the molecular orchestrators of antibody transcytosis in an apical/basolateral orientation. Initial findings obtained using the In-Cell BBB-Trans assay show a significant increase in transcytosis of scFv8D3-conjugated antibodies compared to unconjugated antibodies, as well as verify the requirement of endocytosis pathways in transferrin-receptor-mediated transcytosis of scFv8D3-conjugated antibodies (Figure 1).

Together, the described In-Cell BBB-Trans assay provides a robust methodology for quickly and efficiently validating possible transferrin-receptor-related brain shuttling antibodies in murine cells, as well as provides a platform for delineating the molecular mechanisms behind these processes.

## Experimental Section

### Design, Expression, and Purification of Monoclonal Bivalent Antibodies and Proteins

**Figure 2:**
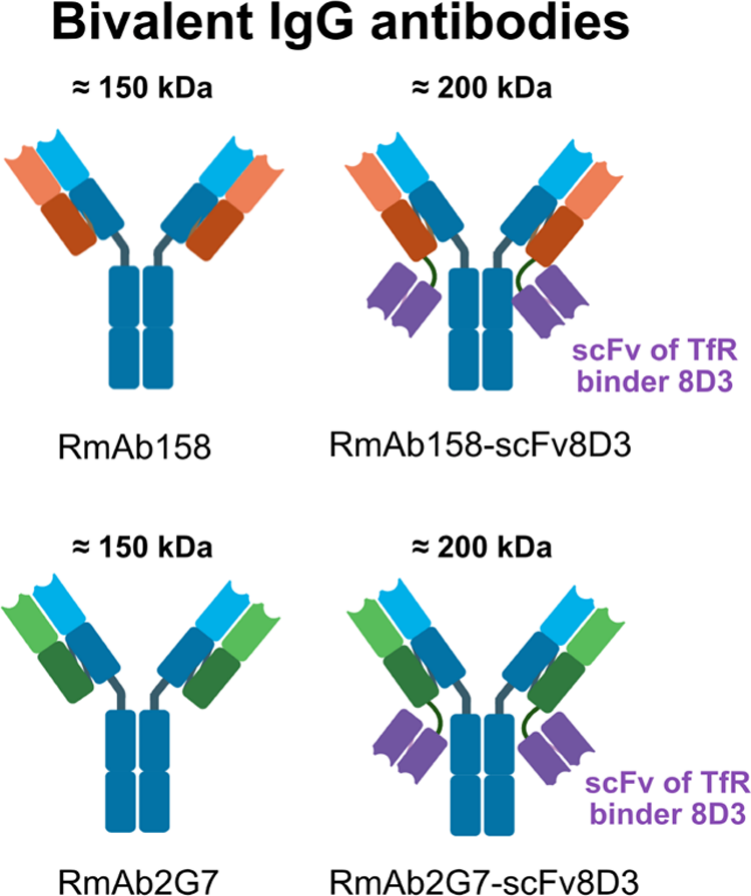
Cartoon representations of the two types of bivalent monoclonal antibodies, with (RmAb158-scFv8D3 and RmAb2G7-scFv8D3) and without (RmAb158 and RmAb2G7) transferrin receptor transporter scFv8D3, used for the In-Cell BBB-Trans assay characterization studies.

The four bivalent monoclonal IgG antibodies and proteins used in the experiments were designed, expressed, and purified according to earlier published work.^6,21^ The RmAb158 monoclonal antibodies selectively bind to Aβ protofibrils,^22^ whereas the RmAb2G7 monoclonal antibodies selectively bind to high-mobility group box 1 (HGMB1) proteins.^23^ In short, the heavy and light chain scFv8D3 transferrin receptor transporter variable region sequence^20^ was connected to the C-terminus of the RmAb2G7 or RmAb158 light chain with in-house designed linkers (APGSYTGSAPG or APGSGTGSAPG, respectively). Figure 2 shows the cartoon representations of the antibody design, showing the location of conjugated scFv8D3 in the modified antibodies.

The four recombinant antibodies were expressed using Expi293 cells (Thermo Fisher) transiently transfected with pcDNA3.4 vectors using polyethyleneimine (PEI) as the transfection reagent. All antibodies were purified on a protein G column (Cytiva) and eluted with an increasing gradient of 0.7% acetic acid. The buffer was exchanged for phosphate-buffered saline (PBS) (Gibco) immediately after elution, and the protein concentration was determined at A280.

### In-Cell BBB-Trans Assay

**Figure 3:**
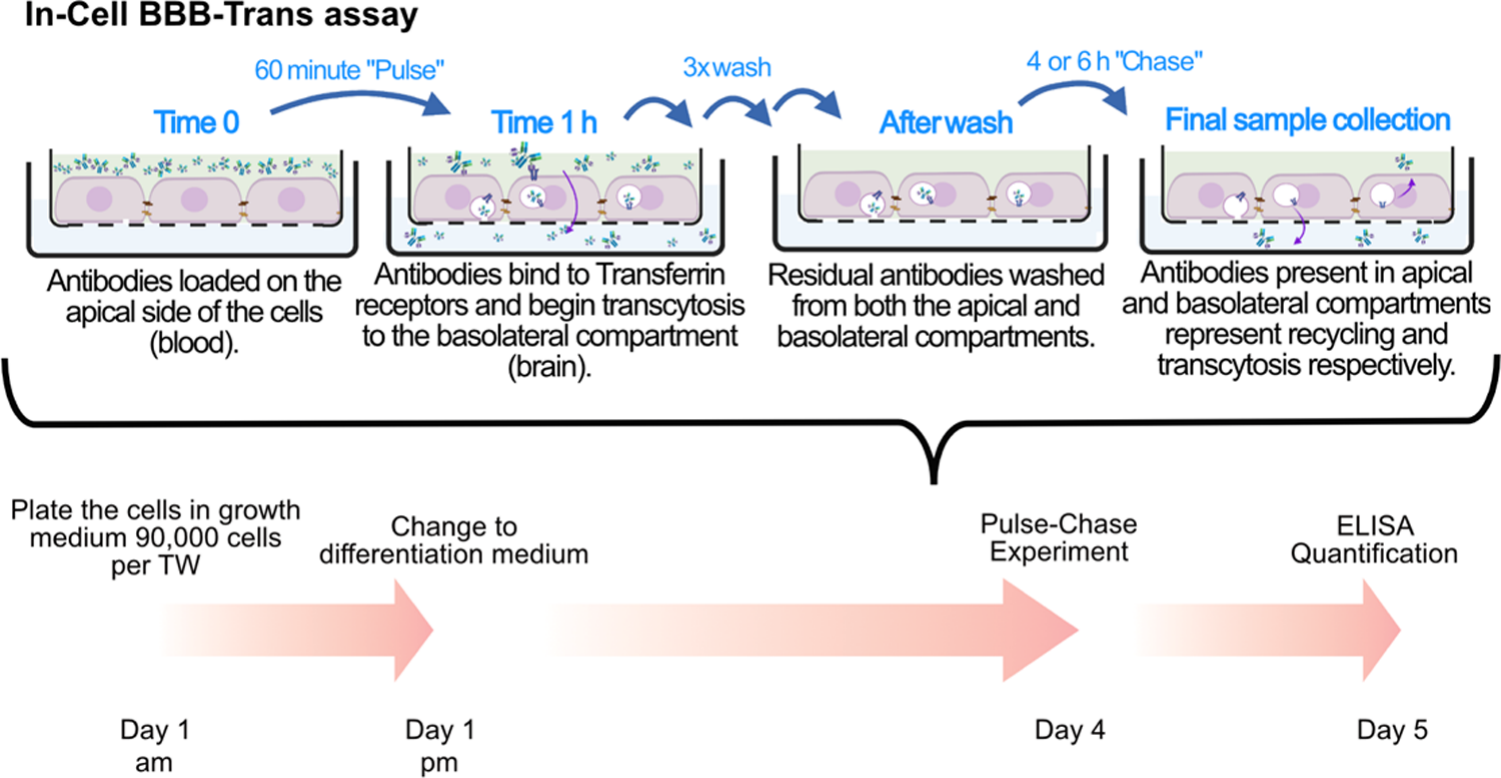
Diagram outlining the different phases of the In-Cell BBB-Trans assay setup, along with a proposed analytical timeline. Time 1 h (pulse), after wash (wash), and final sample collection (chase) are the three phases where media samples were taken for analysis.

A murine cerebral endothelial cell line (cEND) obtained from Applied Biological Materials (passages 15–30 and, as control, 48) was grown on rat tail collagen type I (Sigma—50 μg/mL)-coated 75 cm^2^ culture flasks (Sarstedt) in complete cEND medium (DMEM supplemented with 10% fetal bovine serum (FBS), 1× nonessential amino acids, 1× glutamax, 1 mM sodium pyruvate, and 10 U/mL penicillin/streptomycin—all media and supplements were purchased from Gibco) at 37 °C and 5% CO_2_. For all transcytosis assays, Bio-One Thincert transparent (2 × 10^6^ pores/cm^2^, Cat. no. 662641) and translucent (1 × 10^8^ pores/cm^2^, Cat. no. 662640) polyester (PET) membranes with high-density 0.4 μm pores were used in 24-well cell culture plates (Greiner). Apical chambers of the Greiner hanging inserts were coated with collagen type IV (Fisher Scientific—20 μg/cm^2^) followed by fibronectin (Sigma—20 μg/cm^2^), each incubation lasting for 1 h at 37 °C and 5% CO_2_. The cEND cells were plated at a density of 27 × 10^4^ cells/cm^2^ (9 × 10^4^ cells per PCI) in the apical chamber on day 1 and were refed 4 h later with cEND differentiation medium (2% FBS, 1× nonessential amino acids, 1× glutamax, 1 mM sodium pyruvate, and 10 U/mL penicillin/streptomycin). Optimal media volumes were calculated to be 125 and 800 μL for apical and basolateral chambers, respectively. On day 4, the concentration of each monoclonal bivalent antibody was determined immediately prior to each pulse-chase experiment using a DS-11 spectrophotometer (DeNovix). The Greiner membranes were pulse-incubated apically with 13.3, 133, or 266 nM monoclonal bivalent antibodies in serum-free conditions (no FBS) at 37 °C and 5% CO_2_ for 15 min or 1 h. Aliquots of 75 and 400 μL for the pulse and basolateral chambers were collected to corroborate the starting concentration of the antibodies used and determine the barrier properties of the cEND cells (pulse samples). The monolayers were washed at room temperature in serum-free medium apically (400 μL) and basolaterally (800 μL) three times, with the final wash collected to monitor the removal efficiency of the unbound antibodies (wash samples). Serum-free medium was added to the apical (75 μL) and basolateral (400 μL) chambers. Smaller volumes than the optimal were used to obtain higher concentrations of antibodies in the chase samples, thus making detection with ELISA achievable. The cultures were incubated at 37 °C and 5% CO_2_ for 4 or 6 h, upon which time entire apical and basolateral volumes were collected to assess the recycling and transcytosis of the antibodies into the apical and basolateral chambers, respectively (chase samples). A representation of the In-Cell BBB-Trans assay is shown in Figure 3.

Light cell microscopy images of cEND cells plated on transparent PCI were taken using a Visiscope inverted light microscope (VWR) mounted with a Moticam 1080 BMH digital camera (VWR). Image processing and scale bars were generated using ImageJ software.^24^

### Sandwich ELISA Analysis of Media Samples from the Transcytosis Assay

A 96-well ELISA plate was coated with 1/5000 goat antimouse IgG, F(ab′)_2_ fragment-specific antibody (Jackson ImmunoResearch) diluted in PBS and incubated at 4 °C overnight. The wells were blocked with 1% bovine serum albumin/phosphate-buffered saline (BSA/PBS) for 1 h at room temperature on a 500 rpm shaking platform, followed by washing five times with 0.05% Tween 20/PBS using a Tecan Hydroflex microplate washer. Diluted and undiluted apical and basolateral samples from the transcytosis assay, along with known standard concentrations of monoclonal bivalent antibodies in duplicate (from 0.5 to 128 pM), were added to the wells and incubated for 2 h at room temperature on a 500 rpm shaking platform. The wells were washed as previously described and subsequently incubated with 1/5000 goat antimouse HRP (Sigma) diluted in 0.1% BSA/0.05% Tween 20/PBS for 1 h at room temperature on a 500 rpm shaking platform. Following a final wash cycle, the wells were developed with K-BlueTMB aqueous substrate (Neogen) at room temperature according to the manufacturer’s recommendations using 1 M H_2_SO_4_ to stop the reaction (approximately 5–8 min following the addition of TMB). Absorbance readings at 450 nm were measured immediately using a FLUOstar Omega ELISA plate reader (BMG Labtech), and the data was analyzed using Omega Control (BMG Labtech) and Prism 9 for macOS. Using GraphPad analysis software, interpolation from a standard curve (Sigmoidal, 4PL), based on the concentration of the antibody (ranging from 0.5 to 128 pM), was performed to obtain concentrations of all of the collected samples. The samples were diluted in such a way that they fell within the most linear portion of the curve. Statistical analysis between indicated populations was performed using an unpaired nonparametric Mann–Whitney test, and the minimal accepted significance level was *P* ≤ 0.05.

### Immunohistochemistry of cEND Cell-Plated Translucent PCIs

Following the conclusion of a pulse-chase experiment, cEND-coated translucent PCIs were rinsed two times with PBS and then fixed for 10 min in 4% paraformaldehyde (VWR). Following three further washes with PBS, the PCI membranes were carefully removed using a scalpel blade and mounted vertically in 6% gum tragacanth (Sigma) on a 20 mm cork disc (Thermo Fisher Scientific). The mounted PCI was snap-frozen in dry-ice-cooled isopentane and immediately stored at −80 °C. Using a CryoStar NX70 (Thermo Scientific) cryostat set to −20 °C, eight-micron cryosections were cut and mounted on Thermofrost Plus (Thermo Fisher Scientific) glass cover slides. Individual sections were isolated with a wax pen, rinsed three times with Tris-buffered solution (TBS), and permeabilized for 10 min at room temperature with 0.1% Triton X-100/TBS. Sections were rinsed in 0.05% Tween 20 (wash buffer—diluted in TBS) three times, followed by a 30 min block incubation at room temperature in 10% bovine serum albumin (BSA—diluted in TBS). Sections were rinsed in wash buffer three times, followed by an overnight incubation at 4 °C with primary antibodies diluted in 10% BSA/TBS (1/100 rat antitransferrin receptor (NovusBio), 1/50 goat anti-CD31 (R&D Systems), or 1/100 rabbit anti-Rab5 (Abcam)). Sections were rinsed with wash buffer three times, followed by 1 h room temperature incubation with 1/500 host-targeted fluorescently labeled antibodies (goat antirat Alexa 488, donkey antigoat Alexa 488, goat antirabbit Alexa 555, or donkey antimouse 555 (Thermo Fisher Scientific)). Sections were rinsed with wash buffer three times and mounted with a glass coverslip in Fluoromount-G medium (Thermo Fisher Scientific) supplemented with 100 ng/mL 4′,6-diamidino-2-phenylindole (DAPI). Epifluorescent images were taken using a 10× objective [numerical aperture (NA) 0.30] by an Olympus BX53 fluorescent microscope (Mercury Vapor Short Arc lamp). DAPI was detected using excitation bandwidths 360/370 nm and emission bandwidths 420/460 nm. Alexa 488 was detected using excitation bandwidths 470/495 nm and emission bandwidths 510/550 nm. TIFF images were taken using cellSens Dimension software and further prepared for publication using ImageJ software. Confocal images were taken using a 63× oil objective (NA 1.4–85 nm pixel size) by an LSM700 microscope with Zeiss Zen software. Lasers DAPI 405 (wavelength ranges 420–480 nm), 488 (wavelength ranges < 572 nm), and 555 (wavelength range greater than 560 nm) were detected using photomultiplier tubes (PMTs) using pinhole sizes 60, 69, and 65 μM, respectively. Images were further processed using deconvolution software Huygens Professional (signal-to-noise ratio 30—Scientific Volume Imaging). ImageJ software was used to create an audio video interleave (AVI) movie from individual deconvoluted *z*-stack confocal LSM5 images and also to display Alexa 488 images (TfR) with a false-color look-up table (LUT) to display intensity levels on the membrane cell surfaces.

### Labeling of Antibodies with Iodine-125 (I^125^)

Labeling of the antibodies with I^125^ was performed as previously described.^9^ Equimolar amounts of RmAb2G7 and RmAb2G7-scFv8D3 were each labeled with 8 MBq of I^125^ (Perkin Elmer Inc., Waltham, MA), resulting in a labeling yield of 70%.

### Brain Uptake Studies in Wild-Type Mice

C57Bl/6 male mice (3 months of age) were used in this study. The mice were housed in an animal facility at Uppsala University with free access to water and food and under controlled temperature and humidity. Experimental procedures were approved by the Uppsala County Animal Ethics Board (#5.8.18-13350/17). Mice were intravenously injected via tail vein with a tracer dose of 0.05 mg/kg I^125^-RmAb2G7 (*n* = 6) or I^125^-RmAb2G7-scFv8D3 (*n* = 6). At 2 and 24 h postinjection, three mice were euthanized by transcardial perfusion with 0.9% NaCl. Brains were dissected, and radioactivity was measured using a Wizard 2470 gamma counter (Perkin Elmer Inc., Waltham, MA) as described previously.^9^ Statistical analysis between indicated populations was performed using an unpaired parametric Welch *t*-test, and the minimal accepted significance level was *P* ≤ 0.05. Plasma samples 2 and 6 h postinjection were analyzed using thin-layer chromatography (TLC) to analyze the ratio of I^125^-labeled antibodies vs free I^125^. Briefly, the bottom of a glass jar was filled with 70% acetone. Samples were applied at a baseline on a piece of silica-coated aluminum plate and allowed to dry for approximately 5 min before adding the TLC plate to the solvent-containing glass jar, ensuring that the solvent line was below the sample baseline. When the solvent front had migrated two-thirds of the way up the TLC plate, it was removed from the glass container, allowed to dry for 15 min, and developed underneath an X-ray film in complete darkness. The X-ray film was then measured using a Cyclone Phosphoimager, and the images obtained were analyzed by ImageJ.

### Preparation and Use of Dyngo-4a in the Pulse-Chase Assay

Dyngo-4a (Abcam) was resuspended in sterile-filtered dimethyl sulfoxide (DMSO) to yield a 1000× stock concentration of 30 mM. Thirty minutes prior to pulsing the monoclonal bivalent IgG antibodies on the cEND cell-coated Bio-One Thincert translucent PCIs, a volume of 30 mM Dyngo-4a was added to the apical and basolateral compartments of the cell culture to provide a working concentration of 30 μM. Following preincubation with Dyngo-4a, the apical and basolateral compartments in the pulse and chase phases of the assay (previously described in the Experimental Section) were supplemented with 30 μM Dyngo-4a. Control cultures were performed at the same time, with identical passages of cEND cells, replacing Dyngo-4a with sterile-filtered DMSO (Sigma) at comparable time points.

## Results

### Translucent PCI Matrices Are Optimal for Transcytosis Studies

**Figure 4:**
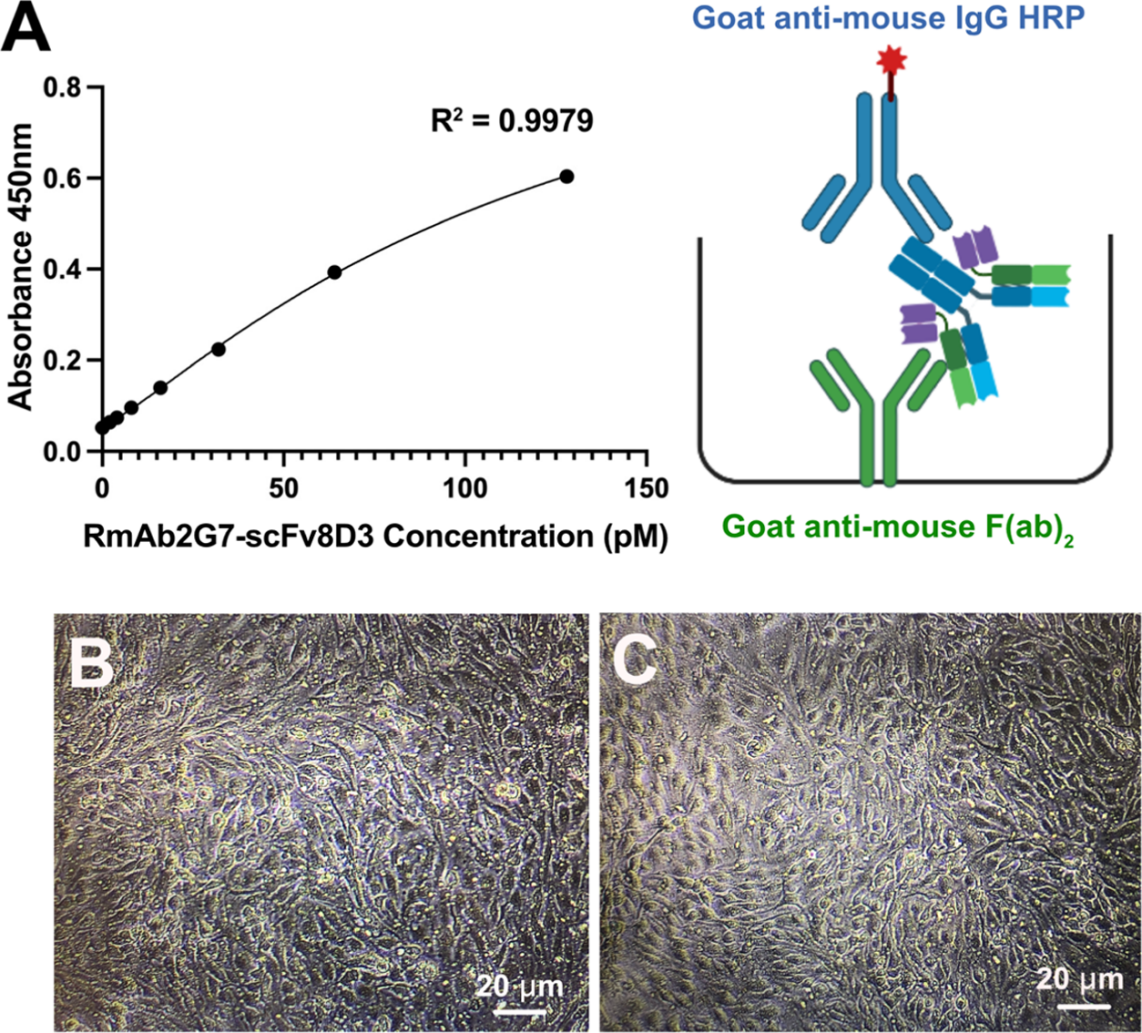
(A) Graphical representation of a routine ELISA standard curve (2–128 pM) for RmAb2G7-scFv8D3 obtained for interpolation of In-Cell BBB-Trans assay media samples, along with a cartoon representation of the sandwich ELISA setup to detect the pulsed monoclonal bivalent antibodies. (B) and (C) Inverted light microscopy images of cEND cells (passage 20) grown on 0.4 μM pore transparent Bio-One 24-well PCI membranes after 3 days in differentiation medium and following a 4 h chase phase in serum-free medium, respectively.

The development of the mouse In-Cell BBB-Trans assay, capable of monitoring transferrin-receptor-mediated transcytosis, required a robust, standardized methodology that allowed for a stringent quality control and washing protocol to quantitatively measure recycling and transcytosis. A Greiner Bio-One PCI-based culture system was chosen as the optimal growth matrix. The In-Cell BBB-Trans assay was employed to load a monolayer of mouse cEND cells (Figure 3), with monoclonal bivalent antibodies conjugated with a transferrin receptor binder (8D3) that has previously been shown to cross the mouse BBB *in vivo*(6,19,20) (Figure 2). Unlike other types of *in vitro* BBB models, the development of a system representing a tight physiologically comparative barrier as seen *in vivo* was not the priority. Instead, the In-Cell BBB-Trans assay relies heavily on quantitively assessing apical recycling or basolateral transport of the loaded antibodies. For the In-Cell BBB-Trans assay to function, a rigorous, quantitative quality control was employed to accurately assess the concentration of the antibody pulsed and the quantity of the antibody remaining in the apical and basolateral compartments following the 1 h pulse and washing protocol. To this end, a highly sensitive ELISA protocol was developed that was able to detect mouse IgG antibodies down to 0.5 pM, allowing for a sensitive, quantitative analysis to be performed. Standard curves, ranging from 0.5 to 128 pM, were only used for interpolation of sample concentrations if the R^2^ value was at least 0.95. Figure 4A depicts a standard sigmoidal curve for the RmAb2G7-scFv8D3 antibody and is representative of standard curves derived for sample concentration interpolation for all other antibodies used throughout this study.

Initial studies were carried out on 24-well 0.4 μM transparent pore PCIs (2 × 10^6^ pores/cm^2^), which allowed for visualization of the cEND cell monolayer during the preparatory differentiation phase (Figure 4B) and following the chase phase (Figure 4C) of the experiment. Even though the cell monolayer morphology (size and shape) appeared normal while growing on the PCI matrix prior to and following the chase phase of the experiment, the bivalent antibodies conjugated with and without the 8D3 transporter were undetectable in the basolateral compartment (Supporting Information, Figure S2). The apical recycling was unaffected, however, with a significant increase in the concentrations of RmAb2G7-scFv8D3 in the apical compartments compared to RmAb2G7, whether a 15 min or 1 h pulse was used. In addition, the concentration of the antibody in the apical and basolateral pulse and wash samples indicated that the correct concentration of antibody was pulsed, the cells were performing an adequate barrier function and the wash phase was removing unbound/nonloaded antibody from the cell culture system prior to the chase phase. To test whether the lack of basolateral transport was due to the steric hindrance brought about by too few pores or the material present in the transparent PCI membrane (2 × 10^6^ pores/cm^2^, Cat. no. 662641), a structurally identical 0.4 μM translucent pore membrane (1 × 10^8^ pores/cm^2^, Cat. no. 662640), containing 50 times more pores, was used in the In-Cell BBB-Trans assay. Using the translucent PCIs definitely improved the basolateral transport of the antibodies (Supporting Information, Figure S2) but not a significant increase in the concentration of RmAb2G7-scFv8D3 in the basolateral chase compartments compared to RmAb2G7. Based on these results, it was decided that the translucent PCI membranes would be used for further characterization studies in the In-Cell BBB-Trans assay.

### Monolayer of cEND Provides a Barrier against Antibodies

**Figure 5:**
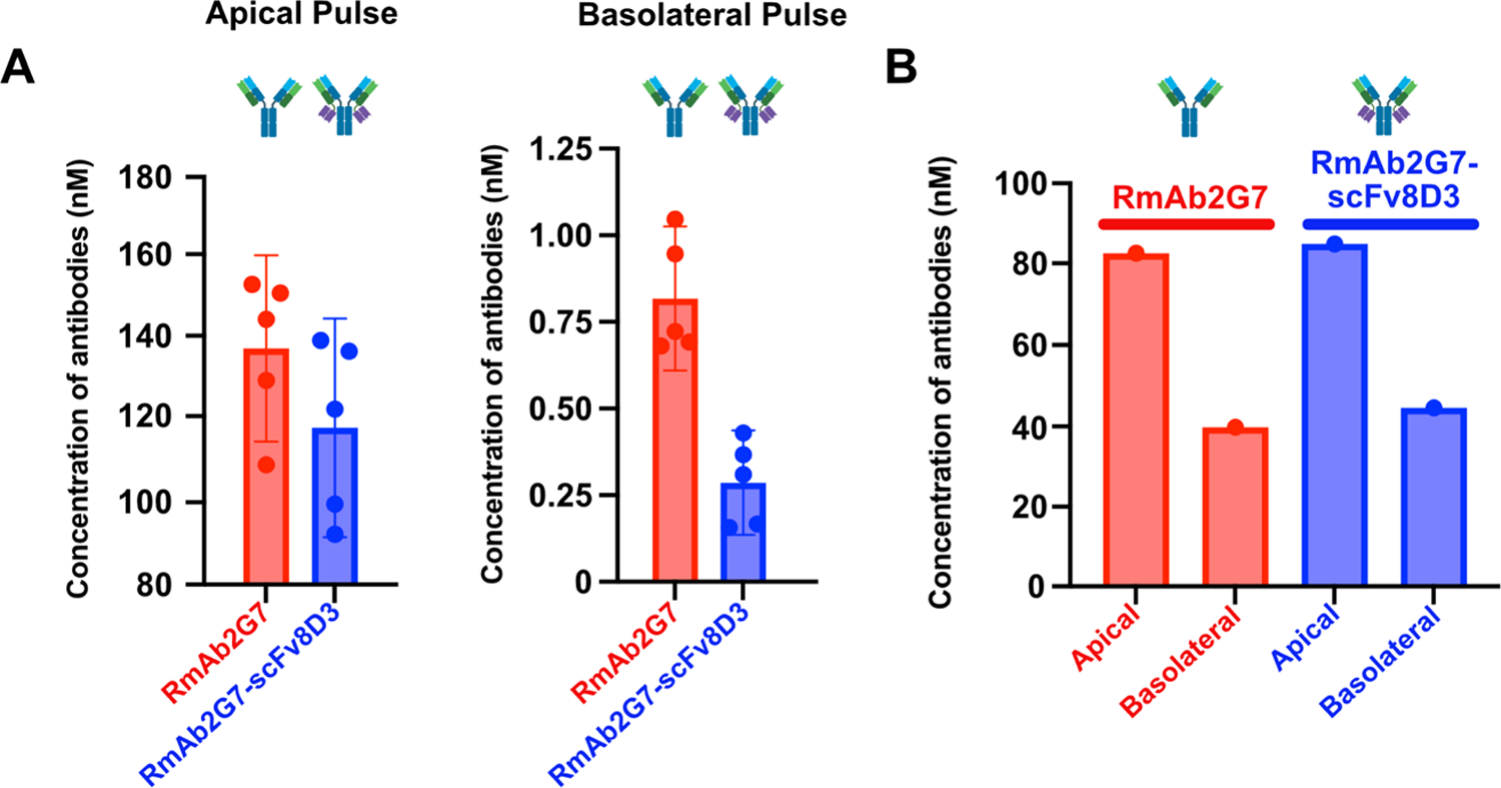
(A) Graphical representation of average antibody concentrations found in the apical and basolateral pulse compartments of cEND cells (passage 20) plated on 0.4 μM translucent pore Bio-One 24-well PCI cultures, following a 1 h “pulse” with either 133 nM RmAb2G7 or RmAb2G7-scFv8D3 monoclonal bivalent antibodies. Six PCIs were used for each pulsed antibody condition. The error bars represent 95% confidence intervals. (B) Graphical representation of antibody concentrations found in the apical and basolateral pulse compartments of 0.4 μM translucent pore Bio-One 24-well PCIs, following a 1 h pulse of either 133 nM RmAb2G7 or RmAb2G7-scFv8D3 monoclonal bivalent antibodies. One PCI was used for each pulsed antibody condition.

As mentioned earlier, the main priority of the In-Cell BBB-Trans assay was to create a standardized model of apical recycling and basolateral transcytosis rather than creating an impermeable, physiologically comparable barrier. However, it was essential that the *in vitro* model does not allow everything to escape through the monolayer of cEND cells during the pulse phase of the assay. Multiple repetitions of the In-Cell BBB-Trans assay revealed that a large proportion of the pulsed antibody concentration remained within the apical compartment of the PCI, whereas around 1–5% of the original antibody concentration travels to the basolateral compartment (Figure 5A).

To assess the barrier capabilities of the cEND monolayer, the same experiment was repeated without the addition of the cEND cells to the PCI. In the space of the 1 h pulse, approximately 30–33% of the original antibody concentration diffused to the basolateral compartment, representing an approximate 10- to 20-fold increase in transport across the PCI membrane compared to when a plated monolayer of cEND cells is present (Figure 5B). This result provides evidence that the cEND cells limit the transport of large macromolecules across the PCI and that at least 95% of the antibodies are in the apical compartment following a 1 h pulse.

### Transverse, High-Resolution Images through the cEND Monolayer Provide Greater Analytical Capabilities

**Figure 6:**
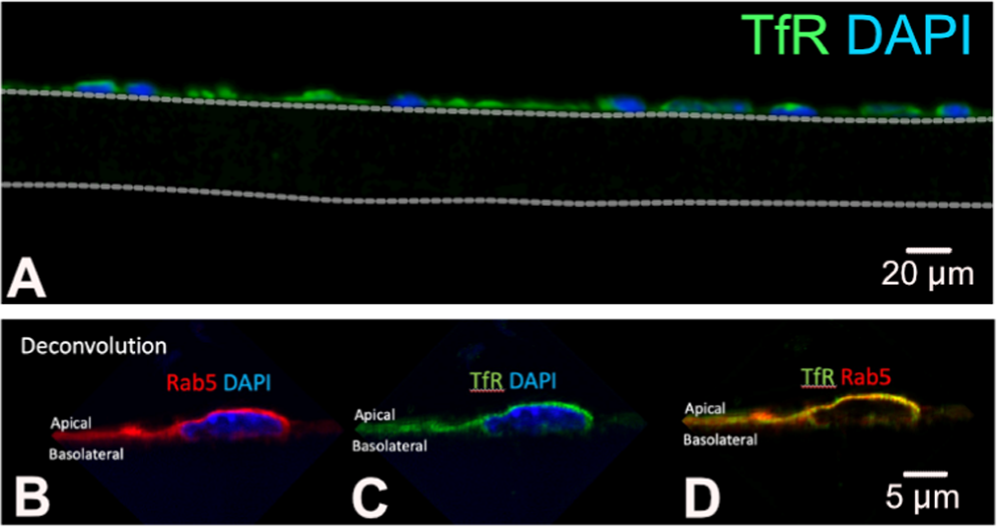
(A) and (B) Photomicrograph representations of 8 μM sections of cEND cells grown on a 0.4 μM pore translucent Bio-One 24-well PCI, immunofluorescently labeled with transferrin receptor (green), Rab5 (red), and DAPI (blue). The image in (A) was taken using an inverted epifluorescent microscope, with the dotted white lines demarcating the upper and lower boundaries of the PCI membrane. The images in (C, D) were taken using an inverted confocal microscope and processed using deconvoluting software. Scale bars were added to the images using ImageJ software.

One of the drawbacks of using translucent PCI membranes is the lack of optical transparency, making microscopic viewing of the cells impossible. However, it is possible to mount the cEND-coated PCIs in such a fashion that ultrathin transverse cryosections can be made through the cells, removing the PCI optical barrier and providing cross-sectional cellular images in an apical-to-basolateral orientation. One can immunofluorescently label the cell sections, highlighting proteins likely involved in the processes of transferrin-receptor-mediated transcytosis. Figure 6A shows a basic epifluorescent image of a cEND monolayer section, colabeled with the transferrin receptor (green) and DAPI (blue) to visualize the nuclei, clearly showing a monolayer of cEND cells assembled on top of the PCI membrane in a monolayer.

Higher-resolution deconvoluted confocal images can further delineate proteins likely involved with transcytosis and their location within the cell. This is exemplified in Figure 6C, where a polarized expression of the transferrin receptor (green) can be seen localized to the apical membrane of the cell, with minimal TfR expression found on the basolateral side. LUT-color intensity analysis further confirms a higher expression of TfR on the apical membrane of the cEND cells compared to the basolateral aspect (Supporting Information, Figure S1). The early endosome marker Rab5 (red) can also be localized in the vicinity of the transferrin receptor (Figure 6B), along with some overlapping of expression (Figure 6D). Supporting Information, Movie S1 shows a *z*-stack compilation through cross sections of cEND cells labeled with the endothelial cell marker CD31 (green), where one can visualize the pulsed RmAb2G7-scFv8D3 antibodies (labeled in red) dispersed throughout the cytoplasm and adjacent to the membrane of the cEND cells. In summary, the described immunohistochemistry technique, along with higher magnification/resolution microscopy, can be adapted and applied to the In-Cell BBB-Trans assay setup, allowing for a greater in-depth molecular delineation of the transcytosis-associated pathways.

### In-Cell BBB-Trans Assay Can be Used to Identify Transferrin-Receptor-Mediated Transcytosis

**Figure 7:**
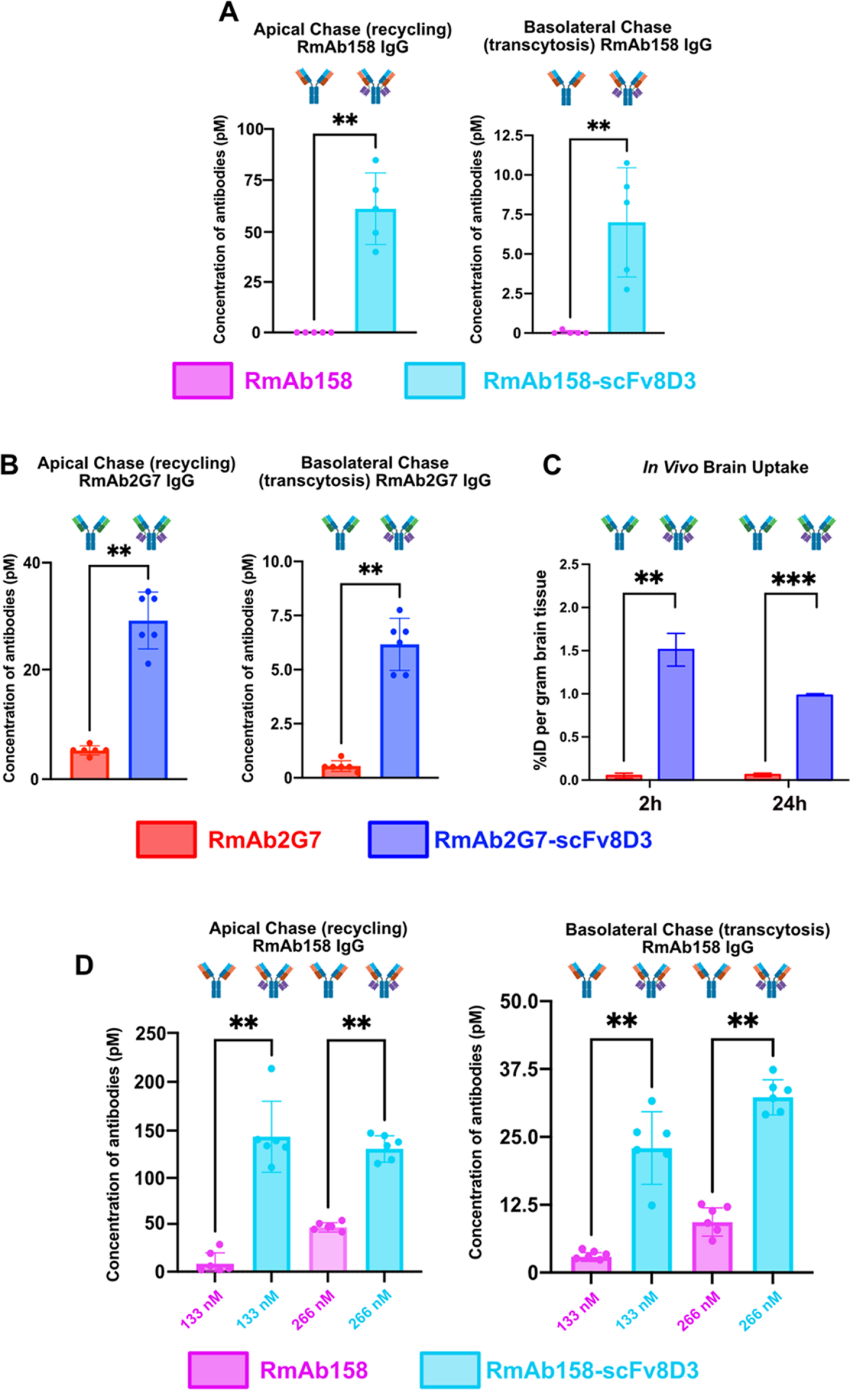
(A) Graphical representation of average antibody concentrations found in the apical and basolateral 4 h chase compartments of cEND cells (passage 18) plated on 0.4 μM translucent pore Bio-One 24-well PCI cultures, following a 1 h pulse with either 13.3 nM RmAb158 or RmAb158-scFv8D3 monoclonal bivalent antibodies. (B) Graphical representation of average antibody concentrations found in the apical and basolateral 4 h chase compartments of cEND cells (passage 15) plated on 0.4 μM translucent pore Bio-One 24-well PCI cultures, following a 1 h pulse with either 13.3 nM RmAb2G7 or RmAb2G7-scFv8D3 monoclonal bivalent antibodies. (C) Comparison of I^125^-RmAb2G7 and I^125^-RmAb2G7-scFv8D3 concentrations in the brain of 3 month old C57Bl/6 wild-type mice 2 and 24 h postinjection. (D) Graphical representation of average antibody concentrations found in the apical and basolateral 6 h chase compartments of cEND cells (passages 29 and 30) plated on 0.4 μM translucent pore Bio-One 24-well PCI cultures, following a 1 h pulse with either 133 or 266 nM RmAb158 or RmAb158-scFv8D3 monoclonal bivalent antibodies. Six PCIs were used for each pulsed antibody condition, except for 13.3 nM RmAb158 and RmAb158-scFv8D3 monoclonal bivalent antibodies (C), where five PCIs were used. The error bars represent 95% confidence intervals. ** Represents a significance level of *P* < 0.01. *** represents a significance level of *P* < 0.001.

Pulsing 133 nM bivalent 8D3-conjugated antibodies to cEND cells grown on translucent PCIs provided strong evidence that transcytosis could be quantified in the In-Cell BBB-Trans assay. To further improve the experimental setup, a lower concentration of antibody was pulsed to reduce the possible cross-linking and endosomal retention of the transferrin receptor.^25^ The optimal concentration of the antibody, which will not result in cross-linking, undoubtedly depends upon the binding affinity of scFv8D3 to the TfR. We first tested monoclonal antibodies that selectively target Aβ protofibrils (RmAb158 and RmAb158-scFv8D3). Figure 7A comprehensively shows a significant increase in apical recycling and basolateral transcytosis in the RmAb158-scFv8D3 pulsed cultures compared to RmAb158. These results closely mimic previously published *in vivo* data by our group,^6^ which showed an 80-fold increased brain uptake following intravenous administration of RmAb158-scFv8D3 in C57Bl/6 mice compared to RmAb158. To show that the process we were observing in our *in vitro* model system was down to the action of 8D3 alone and not some nonspecific pathway based on the antibodies’ selective binding capabilities, we next tested RmAb2G7, an antibody designed to target HGMB1. Once again, Figure 7B shows a very similar outcome when comparing apical recycling and basolateral transcytosis in RmAb2G7-scFv8D3 pulsed cells with those in RmAb2G7. Interestingly, when we performed an *in vivo* brain uptake study with these antibodies in C57Bl/6 mice, we showed comparable results to our *in vitro* data, with a significant increase in brain uptake with RmAb2G7-scFv8D3 compared to RmAb2G7 (Figure 7C). To confirm the veracity of the In-Cell BBB-Trans assay, the experiments were repeated “pulsing” 13.3 nM RmAb2G7, RmAb2G7-scFv8D3, RmAb158, and RmAb158-scFv8D3 for 1 h using a different batch of cEND cells at a different passage and a long chase of 6 h. The data shown in Supporting Information, Figure S3 corroborates the data shown in Figure 7A,B, with a significant increase in apical recycling and basolateral transcytosis in scFv8D3-conjugated antibody pulsed cultures compared to antibodies lacking scFv8D3. These results show that the In-Cell BBB-Trans assay is a robust model that mimics brain uptake of transferrin-receptor-targeted bivalent antibodies *in vivo*. Further studies using elevated concentrations of pulsed bivalent monoclonal antibodies, and an increased chase phase (6 h instead of four), also showed clear transport disparities between antibodies conjugated with and without the scFv8D3 transporter. Figure 7D shows that apical recycling and basolateral transcytosis are significantly higher in RmAb158-scFv8D3 than in RmAb158 at pulsed concentrations of 133 and 266 nM. Looking at the concentration of antibodies present in the final wash samples (Supporting Information, Figures S3 and S4), one can see that only a minute percentage (<0.005%) of the pulsed antibody remains in the apical and basolateral compartments following the wash cycle of the assay, providing further evidence that the increased concentration of antibodies we are seeing in the corresponding apical and basolateral chase compartments (Figure 7A,B,D) is a result of the antibodies that have entered the cells during the pulse phase of the assay. Data shown in Supporting Information, Figures S3 and S4 is representative of antibody concentrations detected in the washes of all performed In-Cell BBB-Trans assays. In addition, note the elevation of the RmAb158 levels in both the 133 and 266 nM pulsed cultures (Figure 7D) compared to 13.3 nM pulsed culture (Figure 7A), which could indicate that the antibody is migrating across the cEND monolayer via alternate nontransferrin receptor-mediated transport pathways at higher concentrations. With this said, it should be noted that the chase times for the 13.3 nM experiments were different from those for 133 and 266 nM experiments (4 and 6 h, respectively), making it difficult to directly compare these experiments. Based on these findings, to ascertain the antibodies’ ability to undergo specific transferrin-receptor-mediated transcytosis, it would be ideal to use the lower pulse concentration of 13.3 nM. Supporting Information, Figure S5 shows that 2 and 6 h after the injection of I^125^- labeled antibodies in the tail vein, the antibodies are still intact and very little free iodine can be detected.

### Transferrin-Receptor-Mediated Transcytosis Requires Endocytosis

**Figure 8:**
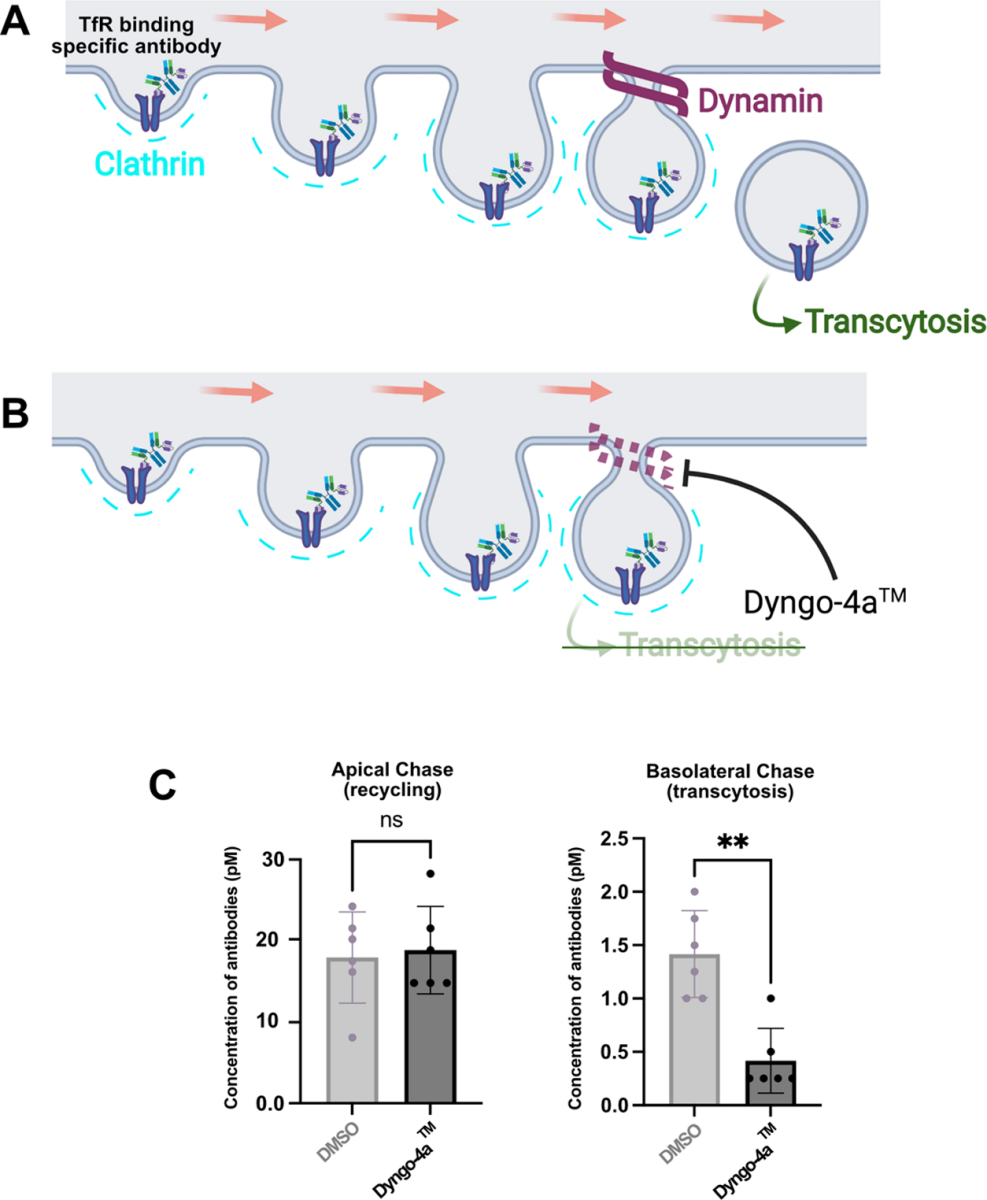
(A) and (B) Cartoon representations of canonical endocytosis of the transferrin-receptor-bound monoclonal bivalent antibodies, conjugated to scFv8D3, in the absence or presence of the endocytosis inhibitor Dyngo-4a, respectively. (C) Graphical representation of average antibody concentrations found in the apical and basolateral 4 h chase compartments of cEND cells (passage 29) plated on 0.4 μM translucent pore Bio-One 24-well PCI cultures, following a 1 h pulse of 13.3 nM RmAb2G7-scFv8D3 monoclonal bivalent antibody supplemented with DMSO (carrier) or 30 μM Dyngo-4a 30 min “prepulse”, pulse, and “chase” phases of the In-Cell BBB-Trans assay. Six PCIs were used for each pulsed antibody condition. The error bars represent 95% confidence intervals. ** represents a significance level of *P* < 0.01.

Transferrin is an iron-binding protein that binds to the transferrin receptor and enters the cell through endocytosis-mediated processes.^26^ The precise mechanistic details on the role of endocytosis in transferrin-receptor-mediated transcytosis of administered antibodies remains to be elucidated and is still up for debate.^27−29^ To gain insight into whether endocytosis is necessary for transcytosis of pulsed 8D3-conjugated antibodies, an endocytosis inhibitor Dyngo-4a (30 μM) was added to the cEND monolayer 30 min prior to and during the pulse phase, as well as during the duration of the chase phase of the In-Cell BBB-Trans assay. As illustrated in Figure 8A,B, Dyngo-4a works by inhibiting dynamin, a GTPase essential for completing endocytosis in eukaryotic cells.^30^ Interestingly, as shown in Figure 8C, when Dyngo-4a was added to the culture, the apical recycling of RmAb2G7-scFv8D3 was unaffected, whereas the level of basolateral transcytosis was significantly reduced compared to cells treated with the DMSO carrier. These results primarily indicate that when endocytosis is inhibited, the antibody bound to the transferrin receptor cannot be internalized, resulting in reduced transcytosis. As the antibody-bound transferrin receptor cannot be internalized, the results also indicate that the apical recycling we are observing in our In-Cell BBB-Trans assay is more likely due to antibodies being released from the transferrin receptor on the surface of the cell during the chase phase of the assay, rather than transferrin-receptor-bound antibodies entering the cells and being recycled to the apical compartment using the canonical transferrin receptor recycling pathways.^26^

### Higher cEND Passages Reduce the Sensitivity but Not the Specificity of the *In Vitro* Assay

**Figure 9:**
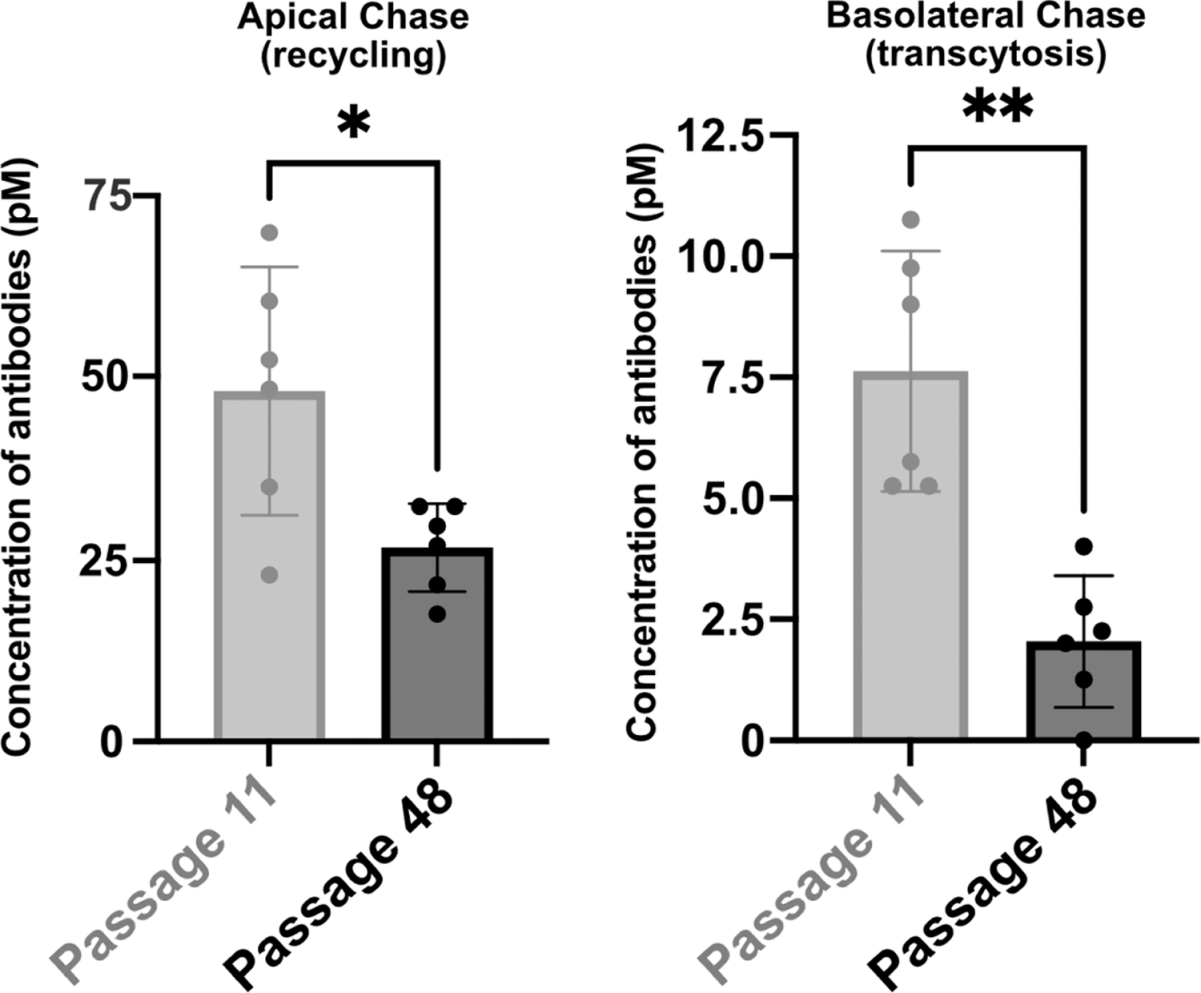
Graphical representation of average antibody concentrations found in the apical and basolateral 4 h chase compartments of Passage 11 and 48 cEND cells plated on 0.4 μM translucent pore Bio-One 24-well PCI cultures, following a 1 h pulse of 13.3 nM RmAb2G7-scFv8D3. Six PCIs were used for each pulsed antibody condition. The error bars represent 95% confidence intervals. * represents a significance level of *P* < 0.05. ** represents a significance level of *P* < 0.01.

To ensure that the In-Cell BBB-Trans assay is sustainable and can be used after multiple passages of cEND cells, the In-Cell BBB-Trans assay was performed using cEND cells at lower and higher passages to compare the apical recycling and basolateral transcytosis of an 8D3-conjugated bivalent monoclonal antibody. Figure 9 shows that both apical recycling and basolateral transcytosis are significantly reduced in passage 48 cEND cells compared to passage 11.

These results explain the discrepancy observed between the absolute concentrations of apical recycling and basolateral transcytosis levels in the In-Cell BBB-Trans assay results shown in Figures 7 and 8, as different cEND passages were used to run each experimental set. This is clearly exemplified when comparing the reduced apical recycling and basolateral transcytosis levels of 13.3 nM RmAb2G7-scFv8D3 pulsed passage 29 cEND cells in Figure 8 with those of 13.3 nM RmAb2G7-scFv8D3 pulsed passage 15 cEND cells in Figure 7B. In summary, even though the level of apical recycling and basolateral transcytosis sensitivity is reduced as the passage number increases, it is still possible to run comparative transferrin-receptor-mediated transport analysis to ascertain transcytosis efficacy, as long as the assay is completed using the same passage of cells.

## Discussion

The need for designing therapeutic strategies that can safely cross the BBB to treat different neurological maladies is of the utmost importance, and coinciding with the development of these strategies, robust methodologies must be in place to preclinically test any promising candidates. The requirement for preclinical testing of any biologic therapy cannot forgo *in vivo* systems when evaluating safety and efficacy. However, aside from the cost and inherent difficulties of planning and carrying out animal experimentation to test biopharmaceutical treatment strategies, it is our duty to seriously consider the reduction, replacement, and refinement protocols for all aspects of preclinical *in vivo* testing.^31^ Furthermore, *in vitro*-based BBB models are not hampered by the behavioral or systemic effects that drive BBB disruption in *in vivo* models, making it easier to define and identify key cellular/molecular players, targets, and regulators of transport across the BBB.^32^ In an *in vitro*-based system, a minuscule amount of material is needed to perform experiments compared to *in vivo* studies, which is an additional benefit. With this said, the implementation of simple *in vitro* systems that can test multiple biologics and physiological conditions would definitely provide a good foundation to start with when assessing the molecular efficacy of BBB penetration.

We have developed an In-Cell BBB-Trans assay that can effectively assess the BBB penetrance ability of large IgG antibodies using transferrin-receptor-mediated transcytosis pathways. The methodology of using PCI systems to monitor BBB transport is not a novel concept. However, as amazing as they are, a large proportion of these *in vitro* systems rely on creating a physiological like-for-like model, focussing heavily on creating a cellular barrier that does not allow even the smallest of molecules to penetrate it.^33^ The main ideology of the In-Cell BBB-Trans assay differs somewhat by focussing more on the removal of unbound or background pulsed antibodies while maintaining the cells in a minimally disturbed and healthy state, which is conducive for their ability to perform physiological tasks such as transcytosis of bound antibodies. With that said, the cell’s ability to act as a formidable barrier is not completely forsaken, and we have shown that a monolayer of cultured cEND cells reduces the transport of the antibody from the upper apical chambers of the PCI system into the lower basolateral chambers (Figure 5A,B). Using this simple 24-well PCI system, which lends itself to testing multiple targets and repetitions at any one time, we can definitively show the specific transport of modified bivalent antibodies conjugated to the transferrin receptor binder scFv8D3 when compared to unmodified antibodies (Figure 7A,C,D). In addition, we also show similar outcomes to the In-Cell BBB-Trans assay when testing these antibodies using *in vivo* brain uptake studies (Figure 7C and ref (6)). Even though the precise comparative *in vitro* and *in vivo* mechansitic pathways behind the transport of scFv8D3-conjugated antibodies remains to be elucidated, these results indicate that the In-Cell BBB-Trans assay follows the brain uptake of bivalent TfR- binding antibodies *in vivo* and can be employed as a more translatable model system in terms of drug development and preclinical settings. Furthermore, there is a hope that the *in vitro* assay we have developed can lead to the reduction and refinement of the *in vivo* burden of testing TfR-mediated BBB-penetrating therapeutic targets.

Even though it is known that canonical transferrin transport into cells occurs via clathrin-mediated endocytosis pathways via the transferrin receptor,^26^ little is known as to whether antibodies that target the transferrin receptor enter in a similar fashion. A previous study using an elegant *in vitro* BBB organoid array system showed that transcytosis of a monovalent antibody targeting the human transferrin receptor is dependent upon clathrin-mediated endocytosis.^34^ We can quantitatively confirm this finding, as the level of transcytosis of RmAb2G7-scFv8D3 is significantly reduced when endocytosis is inhibited in our In-Cell BBB-Trans assay using dynamin inhibitor Dyngo-4a (Figure 8). Even though the translucent PCIs used in our In-Cell BBB-Trans assay do not lend themselves easily to microscopic studies, we have developed a simple methodology for mounting and sectioning cEND cells grown on PCI membranes, allowing immunohistochemical analysis of proteins that are likely pertinent to transcytosis pathways. Along with the In-Cell BBB-Trans assay, this technique provides a transverse view of the cell monolayer, making the identification of molecular markers that could be important for transcytosis easier since it identifies the expression in relation to the apical and basolateral orientation of the cell (Figure 6, Supporting Information, Figure S1 and Movie S1).

The caveat of using *in vitro* culture systems is the progressive changes that can occur at a cellular level as the passage number increases. A way around this problem is to use embryonic stem cells (ES) and induced pluripotent stem cells (iPS) to produce *in vitro* BBB models, as they can be expanded indefinitely and are capable of differentiating into all of the derivatives of the three germ layers, thus removing the possible downregulation of physiological cellular functions with time.^35,36^ However, the elevated ethical discussion of using such cell systems, along with difficulty in generating homogeneous populations of cells, provides a hindrance to using such cell systems to produce large-scale *in vitro* BBB models. Our described In-Cell BBB-Trans assay relies on cells that can be easily sourced, cultured without the need for specialist media, and used at a range of passages to determine transferrin-receptor-mediated transcytosis. In addition, the entire In-Cell BBB-Trans assay is expedient, taking a maximum of 4–5 days to go from plating the cells on the PCI membrane to obtaining quantitative data ready for analysis. We show that the cEND cells used in our described In-Cell BBB-Trans assay show apical recycling and basolateral transcytosis at elevated passages, albeit at reduced levels compared to lower passages (Figure 9). However, as long as the pertinent positive and negative controls are added to performed studies, elevated passages do not hinder obtaining comparative quantitative results that would help delineate the efficacy of transcytosis through the cEND monolayer.

## Conclusions

We have developed a rapid, standardized, and reproducible In-Cell BBB-Trans assay that is capable of discerning the transcytosis capabilities of bivalent antibodies using transferrin-receptor-mediated transcytosis pathways, uncannily mimicking the findings of brain uptake studies performed in wild-type mice. The cell culture setup can be manipulated to investigate different physiological settings to dissect transport pathways. In short, the In-Cell BBB-Trans assay provides a platform for the preclinical assessment of TfR-mediated passage of therapeutic intervention strategies across the BBB.

## Supporting Information Available

Photomicrographs depicting TfR expression on the cEND cell shown in Figure 6B (Figure S1), graphical representation of average antibody concentrations found in the apical and basolateral 4 h chase compartments of cEND cells (passages 13–20) (Figure S2), graphical representation of average antibody concentrations found in the apical and basolateral wash and chase compartments of cEND cells (passage 13) (Figure S3), graphical representation of average antibody concentrations found in the apical and basolateral wash compartments of cEND cells plated on 0.4 μM translucent pore Bio-One 24-well PCI cultures (Figure S4), and TLC of plasma 2 and 6 h after injection of I^125^ antibodies in the tail vein (Figure S5) (PDF)
AVI movie representing a *z*-stack series of 8 μM sectioned cEND cells grown on a 0.4 μM translucent pore Bio-One 24-well PCI, immunofluorescently labeled with the endothelial cell marker CD31 (green), monoclonal IgG (red), and DAPI (blue) (Movie S1) (MOV)

## Supplementary Material

mp2c00768_si_001.pdf

mp2c00768_si_002.mov
