# Supplementary material for: Standardized Preclinical In Vitro Blood–Brain Barrier Mouse Assay Validates Endocytosis-Dependent Antibody Transcytosis Using Transferrin-Receptor-Mediated Pathways
Source: Mol Pharm. 2023 Feb 21;20(3):1564–76. doi: 10.1021/acs.molpharmaceut.2c00768 (PMC9997753; doi:10.1021/acs.molpharmaceut.2c00768)
Supplement: Supplementary file 1 — mp2c00768_si_001.pdf [file mp2c00768_si_001.pdf]

Supporting Information for Publication

A standardised pre-clinical *in-vitro blood-brain barrier* mouse assay validates endocytosis dependent antibody transcytosis using transferrin receptor-mediated pathways.

Jamie I. Morrison[1*], Alex Petrovic [1], Nicola G. Metzendorf[1], Fadi Rofo [1], Canan U. Yilmaz[1], Sofia Stenler[1], Hanna Laudon[2] and Greta Hultqvist[1*]

1. Institutionen för Farmaci, Uppsala Universitet, Uppsala, 752 37, Sweden.
2. BioArctic AB, Stockholm, 112 51, Sweden.

Corresponding authors *: Greta Hultqvist; Email: greta.hultqvist@farmaci.uu.se; and Jamie Morrison Email: jamie.morrison@farmaci.uu.se

Phone: +46 70 225352

Supporting Information

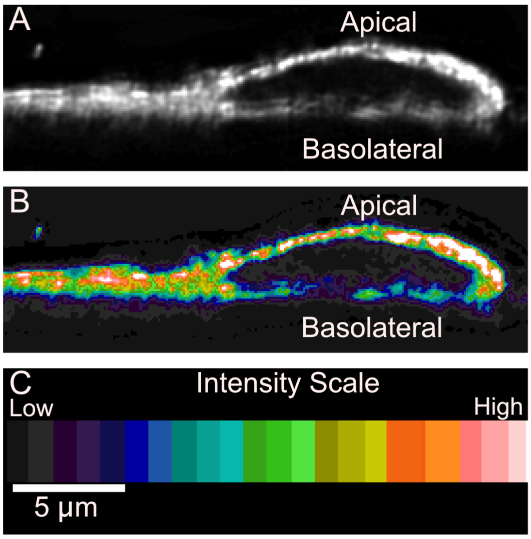

Supplementary Figure 1: Photomicrographs depicting TfR expression on the cEND cell shown in Figure 6B, in greyscale (A) and false-colour LUT (B). An intensity scale, along with a scale bar, can be seen in C. The depicted data shows the differences in intensity of TfR expression, which is clearly polarised with intensities higher on the apical membrane of the cell relative to the basolateral side.

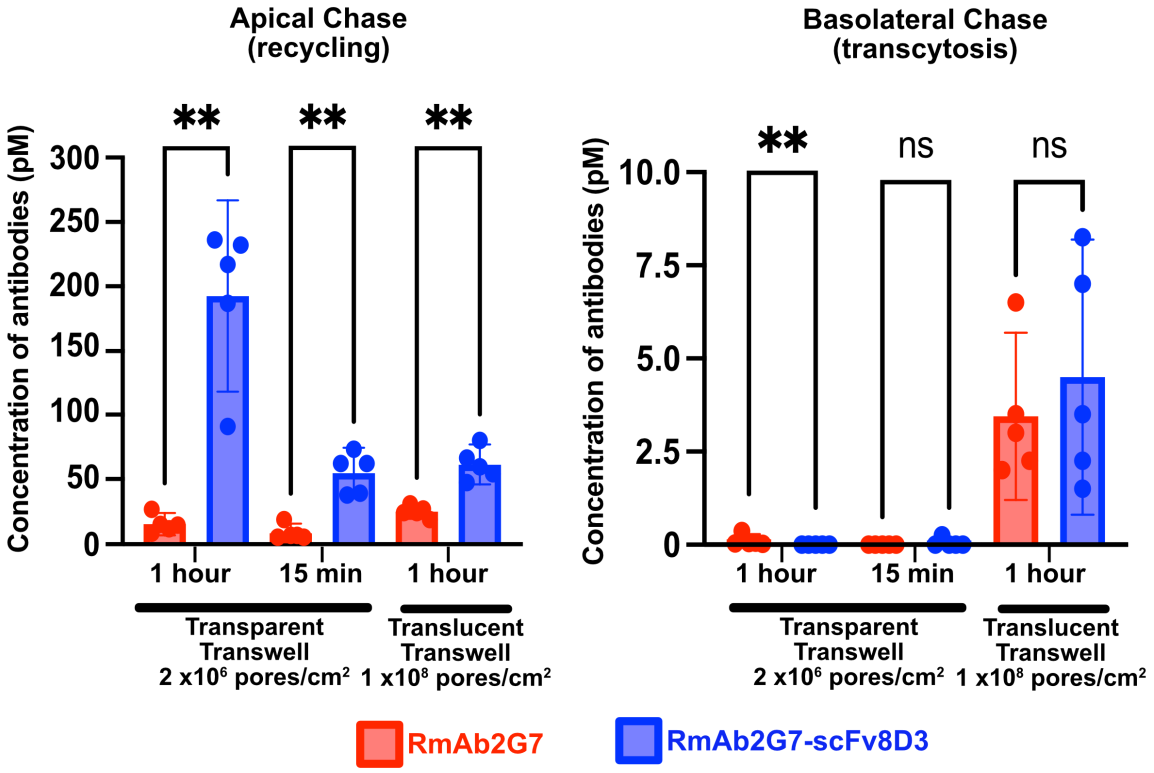

Supplementary Figure 2: Graphical representation of average antibody concentrations found in the apical and basolateral 4-hour chase compartments of cEND cells (passages 13-20) plated on 0.4 µM transparent or translucent pore Bio-One® 24-well PCI cultures, following a 15-minute or one-hour "pulse" of 133 nM RmAb2G7 or RmAb2G7-scFv8D3 monoclonal bivalent antibodies. Six PCIs were used for each pulsed antibody condition. The error bars represent 95 % confidence intervals. n.s. represents a non-significant result. ** represents a significance level of $P<0.01$.

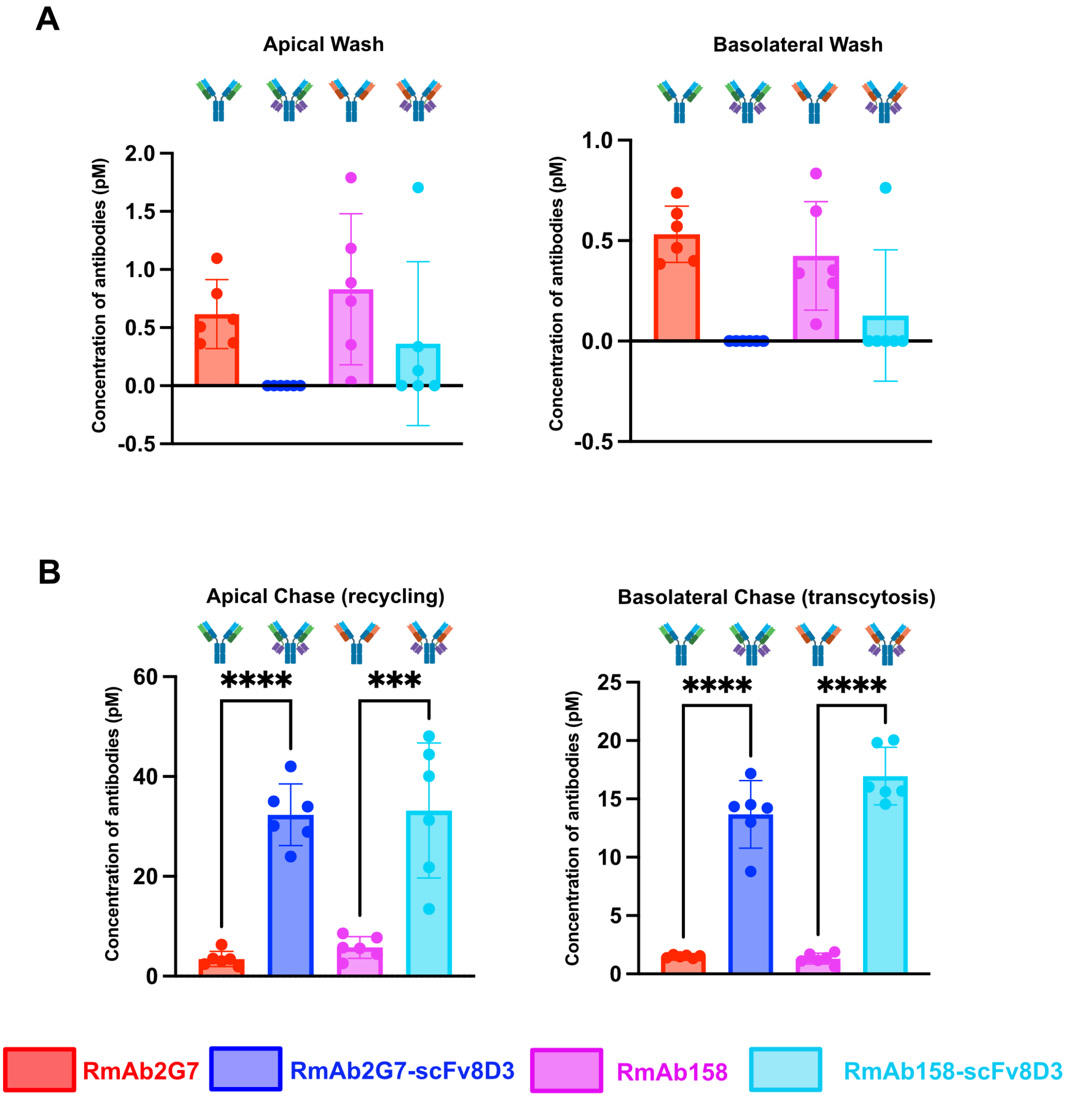

Supplementary Figure 3: Graphical representation of average antibody concentrations found in the apical and basolateral wash (A) and chase (B) compartments of cEND cells (Passage 13) plated on 0.4 µM translucent pore Bio-One® 24-well PCI cultures, following a one-hour "pulse" of 13.3 nM RmAb2G7, RmAb2G7-scFv8D3, RmAb158 or RmAb158-scFv8D3 and a six-hour chase cycle. Six PCIs were used for each pulsed antibody condition. *** represents a significance level of $P<0.001$. **** represents a significance level of $P<0.0001$.

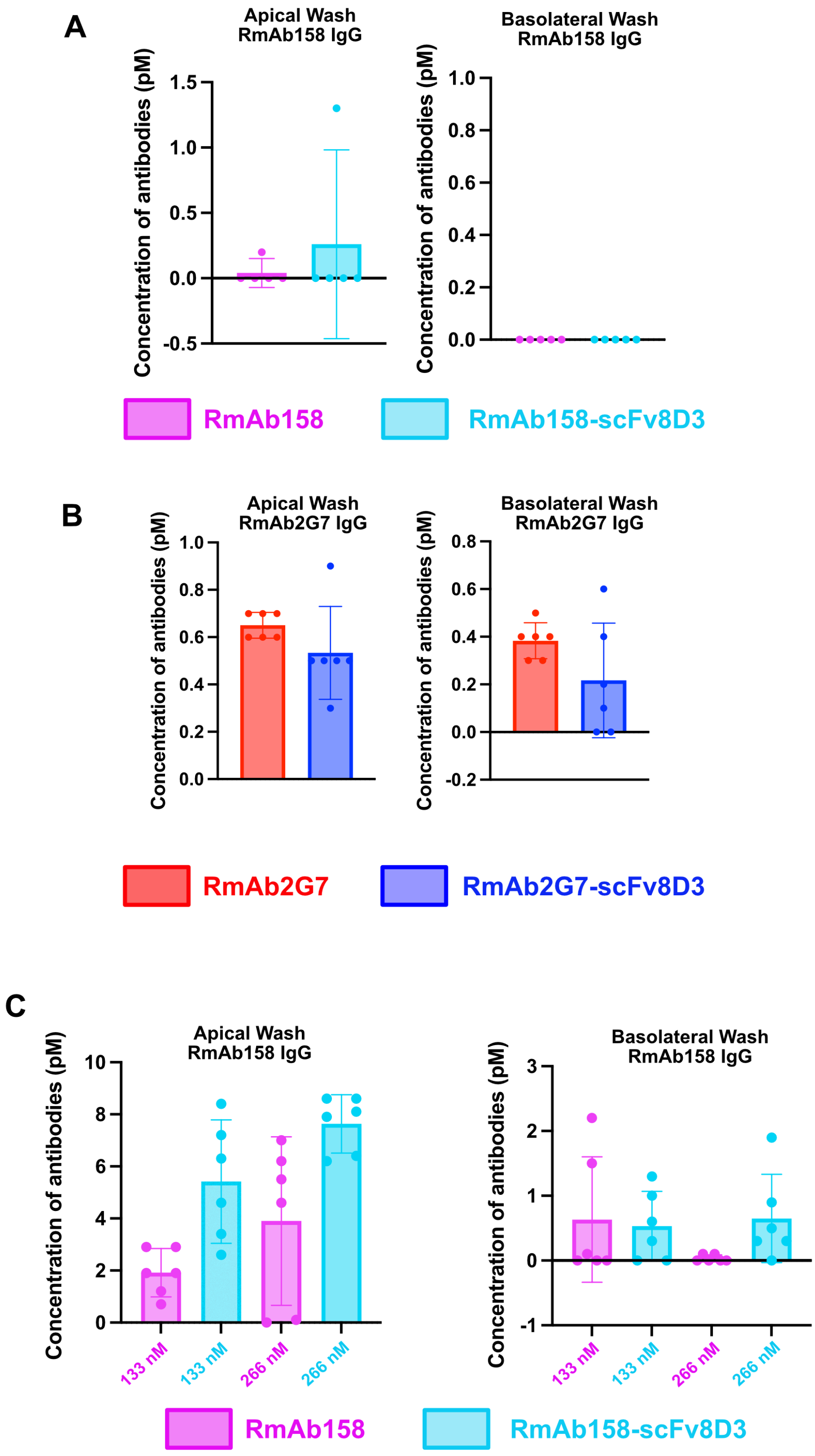

Supplementary Figure 4: Graphical representation of average antibody concentrations found in the apical and basolateral wash compartments of cEND cells plated on 0.4 µM translucent pore Bio-One® 24-well PCI cultures, following a one-hour "pulse" of 13.3 nM RmAb158 or

RmAb158-scFv8D3 (A), 13.3 nM RmAb2G7 or RmAb2G7-scFv8D3 (B) and 133 and 266 nM RmAb158 or RmAb158-scFv8D3 (C) monoclonal bivalent antibodies. The values for the antibody concentration found in the wash samples shown in A, B and C correspond to the chase samples shown in Figure 7A (passage 18), B (passage 15) and C (passage 29 and 30) respectively. Six PCIs were used for each pulsed antibody condition, except A, where five PCIs were used.

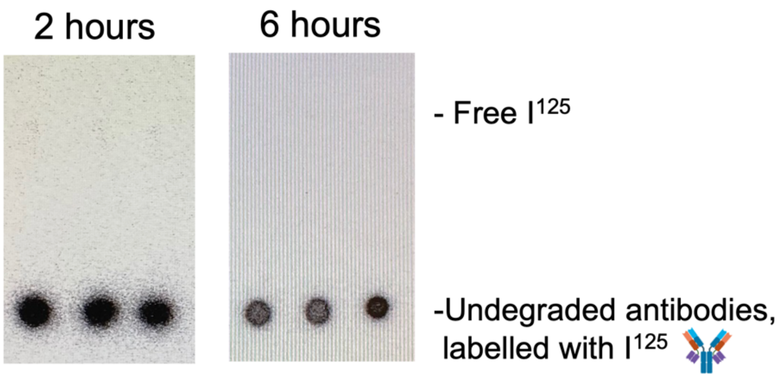

Supplementary Figure 5: TLC of plasma 2 and 6 hours after injection of I^{125} antibodies in the tail vein. In the TLC experiment, I^{125} proteins are separated from free iodine. Almost no free iodine can be detected in the plasma at the analysed time points.

Supplementary Movie 1: An AVI movie representing a z-stack series of 8 µM sectioned cEND cells grown on a 0.4 µM translucent pore Bio-One® 24-well PCI, immunofluorescently labelled with the endothelial cell marker CD31 (green), monoclonal IgG (red) and DAPI (blue). The z-stack images in the movie were taken using an inverted confocal microscope and processed using deconvoluting software. The individual z-stack images were converted to an AVI movie and a scale bar was added using ImageJ software.
